# Mental and physical health in children of women with a history of anorexia nervosa

**DOI:** 10.1007/s00787-024-02393-y

**Published:** 2024-03-13

**Authors:** Sandra Rydberg Dobrescu, Lisa Dinkler, Carina Gillberg, Christopher Gillberg, Maria Råstam, Elisabet Wentz

**Affiliations:** 1 Gillberg Neuropsychiatry Centre, Institute of Neuroscience and Physiology, University of Gothenburg, Gothenburg, Sweden; 2 Department of Medical Epidemiology and Biostatistics, Karolinska Institute, Stockholm, Sweden; 3 Department of Child and Adolescent Psychiatry, University of Glasgow, Glasgow, UK; 4 Department of Clinical Sciences Lund, Child and Adolescent Psychiatry, Lund University, Lund, Sweden; 5 Department of Psychiatry and Neurochemistry, Institute of Neuroscience and Physiology, University of Gothenburg, Gothenburg, Sweden

**Keywords:** Anorexia nervosa, Long-term follow-up, Offspring, Mental health, Physical health

## Abstract

Few studies have investigated the offspring of women with anorexia nervosa (AN). The aim of this study was to examine perinatal status, mental and physical health in the offspring of mothers with a history of AN. Fifty-one individuals with adolescent-onset AN and 51 matched controls (COMP) have been followed prospectively. Presently, 30 years after AN onset, at a mean age of 44 years, female participants who had given birth (n_AN_ = 40, n_COMP_ = 40) were interviewed regarding psychiatric health in their offspring using the Developmental and Well-Being Assessment and the MINI International Neuropsychiatric Interview. In addition, information on the offspring’s perinatal status, psychiatric- and physical health was obtained from the Swedish Medical Birth Register and The Swedish National Patient Register. Data regarding mental and physical health were available for 83 and 86 offspring in the AN and COMP groups, respectively. At birth, all of weight, length, head circumference and ponderal index were significantly reduced in the offspring of mothers with a history of AN. In adolescence, parental interviews indicated an overrepresentation of current psychiatric diagnoses in the offspring of mothers with AN. Compared with the offspring in the COMP group, endocrinological, immune and metabolic disorders were much more common in the offspring of the AN group. In conclusion, a history of AN increases the risk of worse perinatal outcome of the offspring. Later on, in childhood and adolescence, psychiatric and physical morbidity may be overrepresented in the offspring of women with AN.

**Supplementary Information:**

The online version contains supplementary material available at 10.1007/s00787-024-02393-y.

## Introduction

Anorexia nervosa (AN) is one of the most severe psychiatric disorders affecting women of reproductive age. In the acute phase of AN, amenorrhea and infertility are common [1]. According to some studies reproductive functions normalize after weight restoration and recovery from AN [2–4]. Some reports, however, indicate delays in reproduction and reduced fertility rates in individuals with a history of AN [5–7]. An elevated risk of birth and perinatal complications, including higher rates of preterm deliveries, lower birth weight, smaller head circumference, lower Apgar scores and perinatal mortality, compared with controls, has been reported for women with ongoing AN or a history of AN [8–14]. Results from previous studies are partly inconsistent with some studies reporting more favorable perinatal outcomes for women with a history of AN [15, 16].

Parental mental illness is known to be associated with a general increase in psychopathology in the offspring [17–19]. The effect of parental eating disorder (ED) on the offspring’s psychiatric health has received limited attention but available findings indicate that children of mothers with ED have an elevated risk for poorer developmental outcomes including more problems with feeding and eating behaviours, socio-emotional difficulties and psychopathology [20, 21]. A large population-based study showed that 3.5-year-old children of women with a lifetime history of AN had a higher risk of emotional problems in particular, compared with unexposed children [22]. When the children were 7, 10 and 13 years old, they more often had emotional and anxiety disorders compared with controls [23]. Similar findings were presented by Barona, Nybo Andersen and Micali [24] who showed that mothers with lifetime AN were more likely to report emotional problems in their daughters and emotional and conduct problems in their sons. In addition, an increased risk of neurodevelopmental disorders in children of mothers with EDs, especially of those with EDs during pregnancy, has been reported [25]. In contrast, Stein et al. [26] found no differences in general psychopathology between 10-year-old children of mothers with an ED and controls. Moreover, children of mothers with a history of ED are more likely to develop an ED themselves [27]. The influence of parental ED is partly explained by genetics as family and twin studies repeatedly have shown that EDs are strongly familial. Relatives of probands with AN demonstrate that the disorder aggregates in families [28, 29]. Twin studies have shown heritability estimates of AN ranging from 0.48 to 0.74 [30].

Very little is known about the physical health in children of mothers with ED. A handful of studies have indicated that children of mothers with an ongoing ED or with a history of ED exhibit poor weight gain and poor growth development [31, 32]. In severe cases, the aberrant development has been classified as secondary to undernourishment [33].

To summarize, growing evidence supports a history of AN constituting a risk of perinatal complications. Further, an elevated risk of psychopathology in the offspring of individuals with AN has been reported. The main focus of previous research has been the offspring’s early development and less is known about psychopathology in adolescence and young adulthood. To the best of our knowledge, the physical health of the offspring of mothers with AN has so far not been systematically investigated.

Our research group has conducted a prospective longitudinal case–control study of individuals with adolescent-onset AN over the past 30 years. In the present study, based on the knowledge gaps identified above, we aimed at investigating whether children of mothers with AN constitute a risk group for worse outcome with regard to (i) perinatal status, (ii) the prevalence of EDs and other psychiatric disorders, and (iii) the prevalence of physical disorders. We hypothesized that the offspring in the AN group would exhibit a poorer perinatal status and an increased prevalence of psychiatric morbidity and more physical problems compared with the offspring of the control group.

## Method

### Participants

In 1985, all 4291 individuals born in 1970 and attending eighth grade in schools in Gothenburg, Sweden, were screened for AN. The eight-graders completed an ED symptom questionnaire and underwent a physical examination. One of the authors (MR) scrutinized the growth charts and ED questionnaires of all 4291 children and examined in person each case with a suspicion of AN. In total, 25 individuals (23 females, two males) met the criteria for AN in the 1970 birth cohort. One of the girls refused an in-depth examination, leaving 22 females and two males to constitute the *population-based group*. A *population screening group* was also recruited for the study and consisted of 27 adolescents with AN (26 females, 1 male) born in 1969 and in 1971–1977. The *population screening group* was reported to the researchers by the school health service. The groups were combined to constitute the AN group, consisting of 51 subjects (48 women, 3 men) (see [34, 35] for details). All 51 adolescents met the DSM-III-R [36] and the DSM-IV [37] criteria for AN. A comparison group (COMP group) was recruited, the individuals were selected by the school health nurses and were matched for gender, age and school. In the original study physical and psychiatric symptoms were examined in all individuals in the AN and COMP group, and their mothers were interviewed by a psychiatrist concerning family situation, early development, temperament, personality, physical and mental symptoms. The adolescents of the COMP group had no history of ED. Concerning psychiatric comorbidity, at the time of the first examination, 86% percent of the adolescents in the AN group had an axis I diagnosis (other than AN) or depressed mood, compared with 22% of the individuals in the COMP group. The majority of the diagnoses were mood disorders [34].

#### Previous examinations of the sample

The 51 individuals in the AN group and the 51 individuals in the COMP group have previously been examined on four occasions *(AN study 1, AN study 2, AN study 3 and AN study 4)*, at a mean age of 16, 21, 24 and 32 years [16, 34, 35, 38, 39]. There has been no attrition in any of the previous follow-up studies.

#### The present study

All 102 participants in the AN and the COMP groups were contacted and invited to participate in *AN study 5*, 30 years after the onset of AN, at a mean age of 44 years. Four individuals in the AN group (two women, two men) declined participation, leaving 47 participants. All 51 individuals in the COMP group agreed to participate. Data on the male participants were excluded due to their small number, leaving 46 and 48 participants in the AN and COMP group respectively to answer “the general questions about reproduction and offspring” (described in detail below). Participants with offspring were asked if they would agree to take part in interviews regarding their offspring’s health status. Thirty-three females in the AN group and 40 women in the COMP group agreed to participate. In addition, complementary data were obtained from the Swedish Medical Birth Register (SMBR) (see supplementary figure S1).

### Outcome measures

#### Psychiatric health of the mothers

The MINI International Neuropsychiatric interview [40] was used to assess psychiatric disorders, including EDs, in the mothers. The Eating disorder module of the Structured Clinical Interview for DSM-IV-TR Axis I Disorders (SCID-I) [41] and a DSM-5 [42] criteria checklist for feeding and eating disorders was also used to assign ED diagnoses. The Global Assessment of Functioning (GAF) Scale [37] was applied to measure general outcome. Weight and height were measured on the day of the interviews [43].

#### General questions about reproduction and offspring

All subjects were asked three brief questions about reproduction and offspring: (1) whether the mother had any biological/adopted or stepchildren (and, if so, the gender, age and number of children born); (2) whether the participant had any first-degree relatives with underweight or overweight, any eating-related problems, including EDs, and (3) whether their offspring had any severe somatic or psychiatric disorder.

The results presented below is based on data regarding the biological children in the AN and COMP group.

#### Perinatal status measures

The perinatal outcome measures were based on data from the SMBR. The following perinatal variables were selected: gestational age, birth weight, birth length, head circumference, Apgar score (Appearance, Pulse, Grimace response, Activity, Respiration) [44] at 1, 5 and 10 min, and perinatal death (death within six days of birth). The ponderal index (PI), a standard measure for reporting body mass in newborns (kg/m^3^) was calculated. Preterm birth was defined as a dichotomous variable (birth at < 259 days of gestation). Maternal weight and height were recorded when the mother was enrolled at the maternal health care center, usually between week 6–10 of pregnancy, and were used as a measure of pre-pregnancy body mass index.

#### Measures of health and psychiatric problems in the offspring

For 0–4-year-old children, a brief semi-structured interview was conducted, targeting developmental problems in infancy, including sleep, colic, breastfeeding, selective eating, parental concern regarding weight and psychomotor development. The interview was used for the first time in *AN Study 4*, the 18-year follow-up [16]. The interview has not been tested for validity or reliability. The mothers completed the Strengths and Difficulties Questionnaire (SDQ) regarding children 2–17 years old. The SDQ is a brief well-established, well-validated questionnaire used for general mental health screening in children [45] that has been translated into more than 50 languages. The SDQ comprises 25 items that are divided into five subscales: emotional symptoms, conduct problems, hyperactivity, peer problems and prosocial behavior. Each of the items is rated on a three-point Likert scale: “not true”, “somewhat true”, or “certainly true”. The score of each subscale ranges from 0–20; the higher the score, the more symptoms. The prosocial behavior subscale is inverted. The SDQ is completed by parents reporting on the child’s behavior in the past six months. The Swedish adaptation of the questionnaire has been validated, and the following cut-off scores for values above the 90th percentile have been proposed: total difficulties ≥ 14; emotional symptoms ≥ 5; conduct problems ≥ 4; hyperactivity ≥ 6; peer problems ≥ 5; prosocial behavior ≤ 6 [46]. Regarding children younger than 5 years, cut-off scores suggested by Dahlberg, Fält, Ghaderi, Sarkadi, & Salari [47] were used (total difficulties ≥ 12; emotional symptoms ≥ 3; conduct problems ≥ 4; hyperactivity ≥ 5; peer problems ≥ 2; prosocial behavior ≤ 6).

In the study of psychiatric health in offspring at or above age 18 years, the same interview procedure as for the assessment of psychiatric disorders in the mothers was used, i.e. the mother answered the questions concerning their adult offspring. The parent-reported ADHD Rating Scale IV [48] was administered to investigate Attention-Deficit/Hyperactivity Disorder for children at or above age 5 years. The developmental and well-being assessment (DAWBA), a well-validated parental interview, was used to assess psychiatric health in 5–17-year-old children. The instrument generates ICD-10 and DSM-IV diagnoses. The DAWBA has separate sections covering individual emotional, behavioral and hyperactivity disorders, including a section for diagnosing EDs. A web-based version (www.dawba.net) [49] was administered. The timeframe of the interview is the present and recent past; for instance, anxiety disorders are based on the past four weeks while conduct disorders are based on the previous twelve months.

**Fig. 1:**
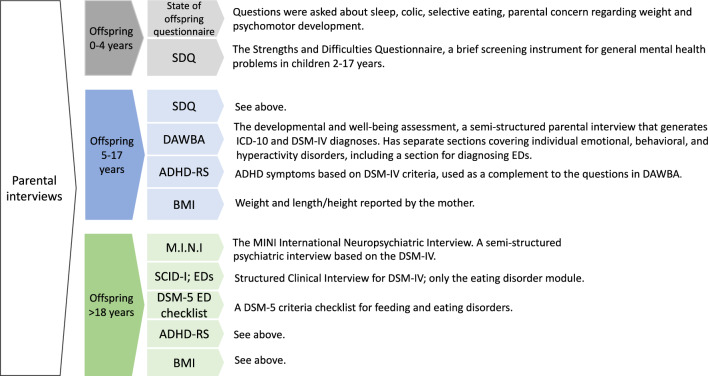
Interview instruments administered to the mothers in the AN and the COMP group regarding the offspring’s health

Interview instruments were selected according to the age of the offspring (see Fig. 1). The interviewer was blinded to the participant’s group status. Before the interview the participant was informed that she should not reveal whether she had a history of AN or not. The interviewer was blinded until all interviews were completed. The majority of the offspring interviews were performed face-to-face, although online video conferences/telephone interviews were performed with twelve of the mothers (AN group, n = 3; COMP group, n = 9).

As a complement to the interviews, the Swedish National Patient Register (NPR) was searched for any psychiatric diagnoses coded according to the International Statistical Classification of diseases and Related health problems, 10th edition [50], codes F00-F99. The NPR covers diagnoses of inpatient care from 1973 and specialized outpatient care from 2001. We had access to data from January 1, 1996, until December 31, 2015. Of the children below 18 years of age, 59 out of 67 (88%) in the AN group, and 57 out of 74 (77%) in the COMP group were found in the NPR.

#### Physical health and body mass index of the offspring

Physical disorders were identified in the NPR using ICD-10 codes for in- and outpatient visits. All mothers of 5–25-year-old offspring were asked to report weight and height of their children, and body mass index (BMI) was calculated and converted to standard deviation scores (SDS) based on the specific age and gender of each child. Four BMI categories were derived: underweight (< -1 SD), normal weight (± 1 SD), overweight (> + 1SD) and obese (> + 2SD).

### Ethics

Data were collected from May 2015 through November 2016. The study was approved by the Regional Ethical Review Board at the University of Gothenburg (398–14). The mothers participated voluntarily after giving their informed consent. Research based on offspring register data was approved for children below age 18 years. For this reason, no register data were extracted for offspring who had turned 18 at the time of the examination of the mother. Informed consent was not collected from the offspring, as they were not participating in the study.

### Statistical analyses

Statistical analysis was carried out by using SPSS, version 28.0. Group comparisons between the mothers in the AN and COMP group were tested mainly with non-parametric tests, due to the data not being normally distributed. The Mann–Whitney test was used for continuous variables and Chi-squared test or Fisher’s Exact test for dichotomous variables. Offspring data were mainly analyzed with Generalized Estimating Equations (GEE) for dichotomous variables, and for continuous variables, a mixed model was used to account for clusters among the women as some contributed to the dataset with multiple births. The significance threshold was set at a p value of < 0.05.

#### Attrition

Total offspring interview attrition applied to seven mothers of 19 children in the AN group and two mothers of four children in the COMP group, leaving 33 and 38 mothers in the AN and COMP group respectively to answer the questions. Of the participating mothers, four children in the AN and three in the COMP group were evaluated only with the SDQ. One of the mothers in the AN group who did not complete the offspring interviews had a current ED. The seven mothers in the AN group who declined the offspring interviews did not differ significantly from the participating mothers with regard to age, BMI and ED duration (mean age: non-participants: 45.3, SD:1.4; participants: 44.4, SD:1.8, p = 0.11; mean BMI: non-participants: 21.4, SD: 1.6; participants: 22.8, SD: 4.6, p = 0.53; mean ED duration in years: non-participants: 6.6, SD: 3.3; participants: 9.7, SD: 7.6, p = 0.30). Additional register data gave us complementary information on the offspring’s status. Combining all sources, at least some data regarding physical and mental health were available on 90.2% and 100% of the offspring in the AN and COMP group, respectively. Thirty-six of the women in the AN group and 32 of the mothers in the COMP group were found in the SMBR, providing data on 82 (88%) and 68 (79%) neonates, respectively. Participants living abroad/giving birth abroad were not found in the SMBR and this applied to four mothers in the AN group and six mothers in the COMP group. Two other women in the COMP group were missing in the SMBR, one had not yet given birth at the time the register data were obtained and one was missing for unknown reasons.

## Results

### Reproduction and clinical characteristics of the mothers

Among the women in the AN and COMP group, 83% (n = 40) in each group had given birth to at least one child (range: AN, 1–5; COMP, 1–4). At the time of assessment, the children in the AN group were between 0–25 years old (mean age = 13.3, SD: 6.0), and the children in the COMP group were between 0–24 years old (mean age = 12.2, SD: 4.9). Table 1 shows demographic and clinical characteristics including reproductive data for the mothers in the AN and COMP groups. In the AN group two women still had AN during pregnancy (one of them had AN during two pregnancies). We found significantly lower BMI and GAF scores among the mothers in the AN group compared with the COMP group at mean age 44 (present study) (Table 1). Psychiatric disorders were significantly more common among the mothers in the AN group.

**Table 1:** Demographic and clinical characteristics, including reproductive data, among the mothers with a history of AN and among the mothers in the COMP group

	Mothers in the AN group	Mothers in the COMP group	*p*
	(N = 40)	(N = 40)	
Age, present study, mean (SD; range)	44.5 (1.8; 38.5–47.6)	44.4 (1.8; 38.4–46.7)	0.72
Age at birth of 1st child, mean (SD; range)	28.8 (6.1; 18–41)	30.4 (4.3; 20–39)	0.19
Children born, in total	93	86	
Gender, children			
Female	46	33	
Male	47	53	
Number of children born, mean (SD)	1.98 (1.33)	1.79 (1.05)	0.45
Parity (n/%)			
1	8 (20.0)	6 (15.0)	
2	21 (52.5)	24 (60.0)	
3	3 (7.5)	8 (20.0)	
4	6 (15.0)	2 (5.0)	
5	2 (5.0)	0	
BMI pre-pregnancy, mean (range)	21.8 (15.2–27.5)^a^	23.6 (18.1–36)^b^	0.069
BMI present study, mean (SD; range)	22.5 (4.2; 16.3–41.5)^c^	24.9 (5.4; 17.2–39.0)	**0.036**
Age at AN onset, mean (SD; range)	14.3 (1.6; 10–17)	n.a	
Duration of AN^d^ in years, mean (SD; range)	5.0 (4.8; 0.9–19)	n.a	
Duration of EDs^d^ in years, mean (SD; range)	9.2 (7.1; 0.9–31)	n.a	
GAF score present study, mean (SD; range)	62.4 (17.7; 30–90)	83.1 (12.3; 50–95)	< **0.001**
Any ED, present study, N (%)	6^c^^,e^ (15.4)	1^f^ (2.5)	**0.048**
AN, present study, N (%)	3^c^ (7.7)	0	**0.045**
Any psychiatric disorder excluding EDs, present study, N (%)	13^c^ (33.3)	3 (7.5)	**0.001**

*AN* Anorexia nervosa, *COMP* Comparison, *SD* Standard deviation, *BMI* Body Mass Index (kg/m^2^), *ED* Eating disorder, *n.a.* not applicable, *GAF* Global Assessment of functioning scale

Statistically significant p values in bold

^a^Based on data from 31 of the mothers in the AN group

^b^Based on data from 27 mothers of the COMP group

^c^Based on data from 39 of the mothers in the AN group

^d^Including several episodes during a follow-up period of 30 years

^e^Anorexia nervosa (n = 3), Other specified feeding and eating disorder (n = 2), Binge-eating disorder (n = 1)

^f^Other specified feeding and eating disorders, night eating syndrome

### Perinatal status of the children

The children’s mean birth weight was lower in the AN than in the COMP group. Birth length and ponderal index were significantly lower and head circumference was significantly smaller in the AN group than in the COMP group (p < 0.05 in all instances). The Apgar scores did not differ between the groups (Table 2). Five and four preterm deliveries were observed in the AN and COMP group, respectively (p = 0.88). One perinatal death had occurred in the AN group.

**Table 2:** Comparison of the perinatal status of offspring of women with a history of AN and offspring of women in the COMP group based on SMBR data

	Children of mothers in the AN group^a^ (N = 82)		Children of mothers in the COMP group^b^ (N = 68)	
	Mean (SD)	Mean (SD)	Difference (95% CI)	*p*
Gestational age (days)	277.8 (13.4)	279.0 (12.6)	−1.2 (−6.6, 4.3)	0.67
Birth weight (g)	3267^c^ (598)	3633^c^ (682)	−365.6 (−653.1, −78.0)	**0.013**
Birth length (cm)	49.4^d^ (2.8)	50.7^e^ (2.6)	−1.3 (−2.5, −0.0)	**0.044**
Ponderal index (kg/m^3^)	26.7^d^ (2.6)	27.8^e^ (2.7)	− 1.1 (−2.0, −0.2)	**0.021**
Head circumference (cm)	34.3 (1.6)	35.3 (1.9)	−1.0 (−1.8, −0.2)	**0.014**
Apgar score at 1 min	8.8 (1.4)	8.7 (1.3)	0.0 (−0.5, 0.6)	0.92
Apgar score at 5 min	9.7 (0.8)	9.7 (0.9)	−0.0 (−0.4, 0.3)	0.89
Apgar score at 10 min	9.9 (0.4)	9.9 (0.4)	−0.0 (−0.2, 0.2)	0.86

Results are based on a mixed model analysis adjusted for clusters within mothers that contributed with multiple births to the dataset. Adjusted means are shown. Statistically significant p values are bolded

*CI* confidence interval

^a^The 82 children were born to 36 mothers with a history of anorexia nervosa

^b^The 68 children were born to 32 mothers with no history of eating disorders

^c^Data missing of 1 child

^d^Data missing of 2 children of the AN mothers

^e^Data missing of 2 children of the COMP mothers

### Early developmental problems, age 0–4 years

**Fig. 2:**
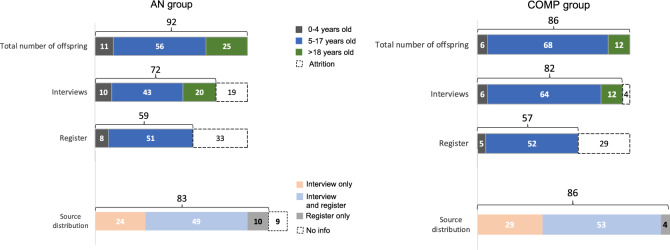
Age and data source distribution in the offspring (N = 92) of the AN group and the COMP group (N = 86) are displayed in this figure. The first row shows the total number of children and the age distribution divided into three groups according to the interview instruments used in the study (0–4 years, 5–17 years, > 18 years old). The second row shows the number of offspring interviews performed and the third row shows the number of offspring found in The Swedish National Patient Register. For ethical reasons, no register data were available for children at or above age 18. The fourth row illustrates the overlap and source distribution of the total offspring sample in the AN and COMP groups

The age distribution of the offspring in the AN group and the COMP group is shown in Fig. 2. Ten children of six mothers in the AN group, and six children of five mothers in the COMP group were included in the brief interview concerning early development. No significant differences regarding regulatory problems (sleep/colic/selective eating) were found in the AN group compared to the COMP group (AN: N = 5; COMP: N = 2; p = 0.63).

### General psychopathology in the offspring (SDQ), ages 2–17 years

The results of the SDQ showed no significant differences between the groups with respect to the total score (AN group offspring mean 6.6 (95% CI: 4.5; 8.6); COMP group offspring mean 7.2 (95% CI: 5.3; 9.0), p = 0.66). The percentage of children rated above the cut-off score for each subscale, in the AN and COMP group respectively, was; emotional problems: 12.8% vs. 7.4%; conduct problems; 8.5% vs. 8.8%; hyperactivity: 6.4% vs. 14.7%; peer problems: 17.0% vs. 8.8%; prosocial behavior: 10.6% vs. 13.2%; total difficulties: 14.9% vs. 11.8% (all differences non-significant). The mean age of the children participating in the SDQ assessment did not differ across groups (AN group: 11.1, SD: 4.4; COMP group: 11.1, SD: 3.5 years).

### Psychiatric diagnoses in the offspring (DAWBA, MINI, SCID-I ED module), ages 5–25 years

The results from the diagnostic interviews are shown in Table 3. The mean age of the participating children in the AN group was significantly higher than in the COMP group (AN group: 14.8 years (SD: 5.0); COMP group: 13.2 years (SD: 4.3), p = 0.045). Current psychiatric disorders based on parental interviews were significantly more common among the offspring in the AN group (Table 3). Lifetime psychiatric disorders based on register and interview data combined were not significantly overrepresented in the offspring of the AN group. Six children in the AN group and one child in the COMP group were diagnosed with a lifetime ED (p = 0.061).

**Table 3:** Psychiatric disorders in the offspring of the AN group and the offspring in the COMP group

	AN offspring n = 59		COMP offspring n = 73		AN vs. COMP
	n	%	n	%	*p*
*Current psychiatric disorders based on interviews*					
Any eating disorder	3^a^	5.1	1^b^	1.4	0.24
Any affective disorder	4	6.8	2	2.7	0.32
Any anxiety disorder	6	10.2	1	1.4	0.076
Any psychiatric disorder	11	18.6	3	4.1	**0.013**

	AN offspring n = 83		COMP offspring n = 86		
	n	%	n	%	*p*
*Lifetime psychiatric disorders, including neurodevelopmental disorders based on registers and interviews*					
Any eating disorder	6^c^	7.2	1^b^	1.2	0.061
Any affective disorder	8	9.6	4	4.7	0.21
Any anxiety disorder	11	13.3	6	7	0.18
Any alcohol dependence	1	1.2	0		
Attention-deficit/hyperactivity disorder	5	6.0	4	4.7	0.69
Autism spectrum disorder	2	2.4	2	2.3	0.97
Attention-deficit/hyperactivity disorder *and* Autism spectrum disorder	1	1.2	1	1.2	
Speech and language disorder	2	2.4	0		
Dyslexia	3	3.6	0		
Other developmental disorders	0		3^d^	3.5	
Any lifetime psychiatric or neurodevelopmental disorder	25	30.1	16	18.6	0.11

Psychiatric disorders are based on the results from the diagnostic parental interviews (DAWBA, MINI, ED module SCID-I) regarding offspring, 5–25 years old. Lifetime psychiatric disorders, including neurodevelopmental disorders, are based on the interviews (MINI, DAWBA and ADHD-RS) combined with retrospective data from the Swedish national patient register concerning offspring 0–25 years old. Affective disorders included major depression and bipolar disorder. Anxiety disorders included panic disorder, agoraphobia, social phobia, specific phobia, obsessive–compulsive disorder, posttraumatic stress disorder, separation anxiety and general anxiety disorder. P values ≤ 0.05 in bold

*AN* Anorexia nervosa, *COMP* Comparison

^a^Eating disorder not otherwise specified; n = 3 (1 male, 2 female)

^b^Anorexia nervosa (n = 1; female)

^c^Anorexia nervosa (n = 1; female), eating disorder not otherwise specified (n = 4; 1 male, 3 female), binge-eating disorder (n = 1; female)

^d^Developmental coordination disorder (n = 2), pervasive developmental disorder (n = 1)

### BMI and physical health in the offspring

**Fig. 3:**
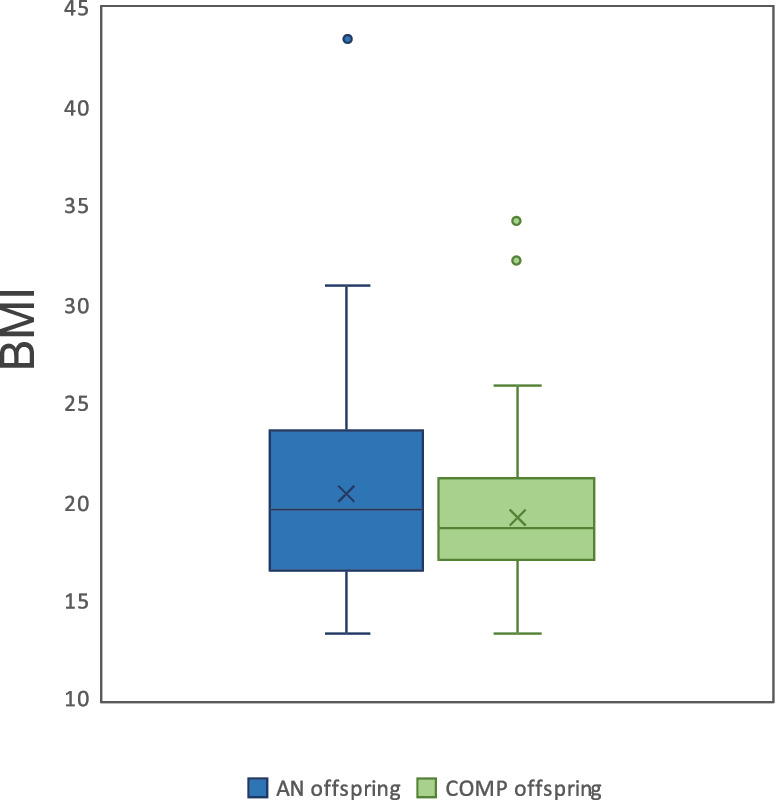
Box plot of the body mass index in the offspring of the AN (N = 59) and the COMP group (N = 76), 5–25 years old

The majority of the children in both groups were within the normal range regarding BMI (Fig. 3). Weight extremes (underweight/overweight/obese) were about equally distributed across groups (25% in AN group; 15% in COMP group, p = 0.17). Endocrine, immune and metabolic diseases were much more common among the offspring in the AN than in the COMP group (p = 0.002) (Table 4). Four of the endocrinological diagnoses related to deviant growth (n = 2) and puberty development (n = 2) (Table 4).

**Table 4:** Physical disorders in offspring in the AN group compared with offspring in the COMP group

	AN offspring (N = 83)	COMP offspring (N = 86)	*p*
**Any endocrine, immune, nutritional and metabolic disease, N (%)**	13^a^ (15.7%)	2 (2.3%)	0.002
Diabetes type 1	0	1	
Diabetes type 2	1	0	
Hypothyroidism	2	0	
Gilbert's syndrome	1	0	
Precocious puberty	1	0	
Disorders of puberty, not elsewhere classified	1	0	
Growth hormone deficiency	1	0	
Short stature	1	0	
Iron deficiency	1	1	
Juvenile arthritis	3	0	
Celiac disorder	2	0	
**Any disease of the digestive system, N (%)**	7 (8.4%)	7 (8.1%)	0.95
Gastritis	0	1	
Functional bowel disease	1	1	
Constipation	6	4	0.51
Melena	0	1	
**Any disease of the nervous system including seizures, N (%)**	6 (7.2%)	3 (3.5%)	0.32
Migraine	2	2	
Epilepsy	1	1	
Febrile seizures	2	1	
Convulsion (unspecified)	2	0	
**Any disease of the respiratory system, N (%)**	15 (18.1%)	8 (9.3%)	0.096
Asthma	11	7	0.11
Pneumonia	4	0	
Chronic lung disease	1	1	
**Other disorders**			
Severe allergy	0	1	
Malformations	5^b^	2^c^	0.23
Supraventricular tachycardia	0	1	
**At least one physical disorder**	28 (33.7%)	21 (24.4%)	0.18

Data regarding physical disorders are mainly based on information from the Swedish national patient register (NPR) (registered offspring: AN; n = 59; COMP; n = 57). Data concerning offspring not found in the NPR (above age 18; AN; n = 18, COMP; n = 12, missing data; AN; n = 5) are based on interview results collected at the time of examination of the mothers

*AN* Anorexia nervosa, *COMP* Comparison

^a^One participant had two conditions; hypothyroidism and disorders of puberty, not elsewhere classified

^b^Including chromosome disorder (n = 1), septal defect (n = 3), plagiocephaly

(n = 1), congenital malformation of face and neck (n = 1)

^c^Congenital deformities of hip (n = 2)

*Excluded diagnoses:* tonsillitis, flu, conditions of the respiratory tract (e. g. acute laryngitis, acute bronchitis), unspecified virus infections, acute appendicitis, gastroenteritis, minor malformations (e.g. prominent ear)

## Discussion

This prospective controlled community-based 30-year follow-up study of adolescent-onset AN aimed at examining perinatal status, psychiatric and physical health in the offspring of mothers with a history of AN. In addition, perinatal status of the children was investigated. At least some data were available on 90.2% of the offspring in the AN group and all of the offspring in the COMP group. Birth weight and length, head circumference and ponderal index were significantly reduced in the offspring of the mothers in the AN group. After the perinatal period, BMI did not differ between the children in the AN and COMP groups. Parental interviews (DAWBA, MINI) indicated a higher prevalence of current psychiatric disorders among the offspring of mothers with a history of AN. The SDQ assessment did however not confirm the presence of more psychiatric symptoms among the children in the AN group. Furthermore, an overrepresentation of endocrinological, immune and metabolic disorders was observed among the offspring in the AN group.

As we had hypothesized, offspring in the AN group exhibited worse perinatal outcomes (lower birth weight and length, lower ponderal index and smaller head circumference) compared with offspring in the COMP group. This finding is consistent with other studies in terms of lower birth weight and smaller head circumference [8, 14]. We did not find an elevated risk of preterm birth or lower Apgar scores in the AN group, which is in agreement with some other reports [15, 51]. The observed reduced birth size in the offspring signals that children of mothers with lifetime AN constitute a risk group for a worse perinatal outcome. Stringer and Furber emphazise the importance that midwifes identify women with ED early in pregnancy and tailor interventions appropriately [52]. We suggest that maternity care routines to identify women with ongoing or previous ED should be established.

Moreover, we had hypothesized that the offspring in the AN group would be at an increased risk of developing psychiatric disorders throughout childhood. Current psychiatric symptoms were not more common in the offspring in the AN group compared with the offspring in the COMP group, using the SDQ. This is in line with findings by Stein et al.[26]. On the other hand, Micali et al. [23] and Barona et al. [24], who also used the SDQ, found an increased prevalence of emotional and conduct disorders in the offspring of mothers with a history of AN. These two studies comprised larger samples than the present study, and we cannot exclude the possibility that our results are due to our sample being underpowered.

The diagnostic interviews concerning current psychiatric disorders in the offspring indicated that psychiatric disorders in general were overrepresented among the offspring in the AN group. The results are consistent with findings by Micali and colleagues [23], showing an overrepresentation of emotional disorders, including anxiety disorders, among children of mothers with EDs. Ten percent of the offspring in the AN group fulfilled the criteria for an anxiety disorder, although the difference failed to reach statistical significance compared with 1.4% of the offspring of the COMP group. Extending the groups to include offspring of all ages (combining register data with interview data), lifetime psychiatric disorders did not differ between the AN and the COMP group.

There was a discrepancy between the diagnostic interview results (DAWBA, MINI) and the SDQ data. The former showed an overrepresentation of current psychiatric disorders among the offspring of the AN group, while the latter did not signal more psychiatric symptoms among the children in the AN group. The difference could reflect the different age ranges of the children involved in the questionnaires versus the interviews; the children rated with the SDQ were younger (mean age: 11.1, age range: 2–17 years) than the offspring in the diagnostic interviews (AN group mean age: 14.8, range: 5–25 years; COMP group mean age: 13.2, range: 5–24 years).

In our sample, six children in the AN group and one adolescent in the COMP group were diagnosed with a lifetime ED, the difference was not statistically significant (p = 0.061). An overrepresentation of EDs among the offspring of mothers with AN has been observed by other groups [28, 53]. Our sample included children in a wide range of ages and a considerable minority of the children in the present study had not yet reached puberty, when the onset of AN and other EDs usually takes place [54]. Although EDs have a genetic component explaining the likelihood of a higher prevalence of EDs in children of mothers with EDs, environmental factors have been put forward as crucial mediators in the expression of underlying predisposition [55]. For instance, nutritional intake during pregnancy may influence the DNA methylation in genes related to growth, metabolism and appetite regulation [56]. Further, the literature implies that a parent with ED impacts on her/his child’s feeding and eating patterns. Stein and colleagues found that during mealtime interactions between mothers with EDs and their children, the mothers expressed more intrusive behaviors and negative emotions towards the infant compared with the interaction between healthy control mothers and their offspring [32]. Our sample was too small to investigate the impact of environmental factors and the mediation of gene expression.

We observed a much higher prevalence of endocrinological, immune and metabolic diseases among the offspring in the AN group. The results need to be replicated using larger samples. The findings are, however, in line with previous reports of an association between EDs and various autoimmune disorders [57, 58]. Furthermore, recent findings have indicated that parental mental illness is associated with an elevated risk of autoimmune diseases in the offspring [59]. In addition, eating disorders have been linked to Paediatric acute-onset neuropsychiatric syndrome (PANS) as one defining feature of some cases with sudden onset of eating restrictions [60]. In individuals with PANS a family history of inflammatory or autoimmune diseases has been reported to be common [61]. Contrary to our expectations, we found no support for impaired growth after the perinatal period in the children born to mothers with a history of AN. Although the ponderal index was lower at birth among the offspring in the AN group, the majority of these children had a normal BMI later on.

## Strengths and limitations

This is the only study that has followed a group of individuals with adolescent-onset AN prospectively, along with a matched comparison group, for as long as 30 years. The sample is community-based and half of the AN cases constituted a total birth cohort. The individuals have been examined on five occasions after their onset of AN. Some larger cohort studies examining psychiatric health in the offspring of mothers with ED have relied on parental questionnaires when studying the offspring’s development e.g. [24]. Our group has, however, interviewed the mothers in person using a semi-structured interview, and therefore the interview data can be considered as reliable [62]. The parental interviews resulted in psychiatric diagnoses in the children according to the DSM-IV, the DSM-5 and the ICD-10. Having access to register data enabled us to obtain complementary information regarding the children. By combining different data sources we obtained at least some information on physical and mental health concerning 90.2% and 100% of the offspring in the AN and COMP group, respectively. The perinatal data covered 88% and 79% of the children in the AN and COMP group, respectively. To the best of our knowledge, this is the first study examining several aspects of health, including perinatal status, psychiatric and somatic morbidity, in the offspring of mothers with a history of AN.

The current study has some obvious limitations. The sample size was relatively small, which is in contrast to some larger register-based studies that have been performed in this area [22, 24, 25]. The small sample size also prevented us from performing gender-stratified analyses. This must be considered a limitation since a previous study found gender differences between children when parents with EDs completed the SDQ [24]. The children were not examined by the researchers. Instead, we had to rely on the parental reports using established and well-validated interviews. Although parent reported weight and height can be considered reasonably accurate regarding children [63], reports of anthropometric measures in adolescent and adult offspring might be less reliable. In order to gain further information, personal assessments of the offspring would have been valuable. Regarding the diagnostic interviews, the offspring in the COMP group were significantly younger than the offspring in the AN group. The children in the former group had therefore had less time to develop psychiatric disorders, including EDs, than the children in the latter group. Another limitation was the wide age range of the offspring groups (from newborn to young adulthood). Further, we had no access to information on psychiatric history of the children’s fathers which limits our understanding of the familial transmission. The phenomenon of nonrandom mating patterns, both within and across psychiatric disorders, might additionally increase the risk of psychiatric disorders in offspring of women with a history of AN as suggested by Nordsletten et al. [64]. Adopted and stepchildren were not included in the study. Due to the small number of adopted children (n = 1), gene-environment interactions could not be investigated.

An additional limitation is that we did not correct for multiple comparison, which increases the risk of type I errors (finding significant results by chance when performing many tests). In order not to miss any potential association we did not adjust for multiple comparisons. Our sample was small, but a larger sample was not possible to collect and therefore the results and conclusions have to be treated as exploratory.

## Conclusions

Our findings confirmed that children of mothers with AN constitute a risk group for worse perinatal outcome. Later on, in childhood, there were few differences regarding psychiatric health between the children in the AN group and the children in the COMP group. However, there was an overrepresentation of current psychiatric disorders among the offspring in the AN group. To our knowledge, this is the first time that an increased prevalence of endocrinological, immune, and metabolic disorders was observed in the offspring of women with a history of AN. Clinically, in maternity care, we suggest that women should be screened for current or previous AN. Women who screen positive for current or previous AN require special consideration in order to reduce negative birth outcomes. In addition, clinicians treating AN patients should be aware that the offspring might have an elevated risk of psychiatric and physical morbidity.

## Supplementary Information

Below is the link to the electronic supplementary material.

Supplementary file1 Fig. S1. Participant flowchart showing original and final sample. Regarding the two females who declined; one had offspring and one had no offspring according to data from the SMBR. AN: Anorexia nervosa; COMP: Comparison; SMBR: the Swedish medical birth register; F: female; M: male. (PDF 27 KB)

## Data Availability

The datasets used and/or analysed during the current study are available from the corresponding author on reasonable request.
