# Supplementary figures and images for: Mental and physical health in children of women with a history of anorexia nervosa
Source: Eur Child Adolesc Psychiatry. 2024 Mar 13;33(10):3481–93. doi: 10.1007/s00787-024-02393-y (PMC11564200; doi:10.1007/s00787-024-02393-y)

## AN group

## COMP group

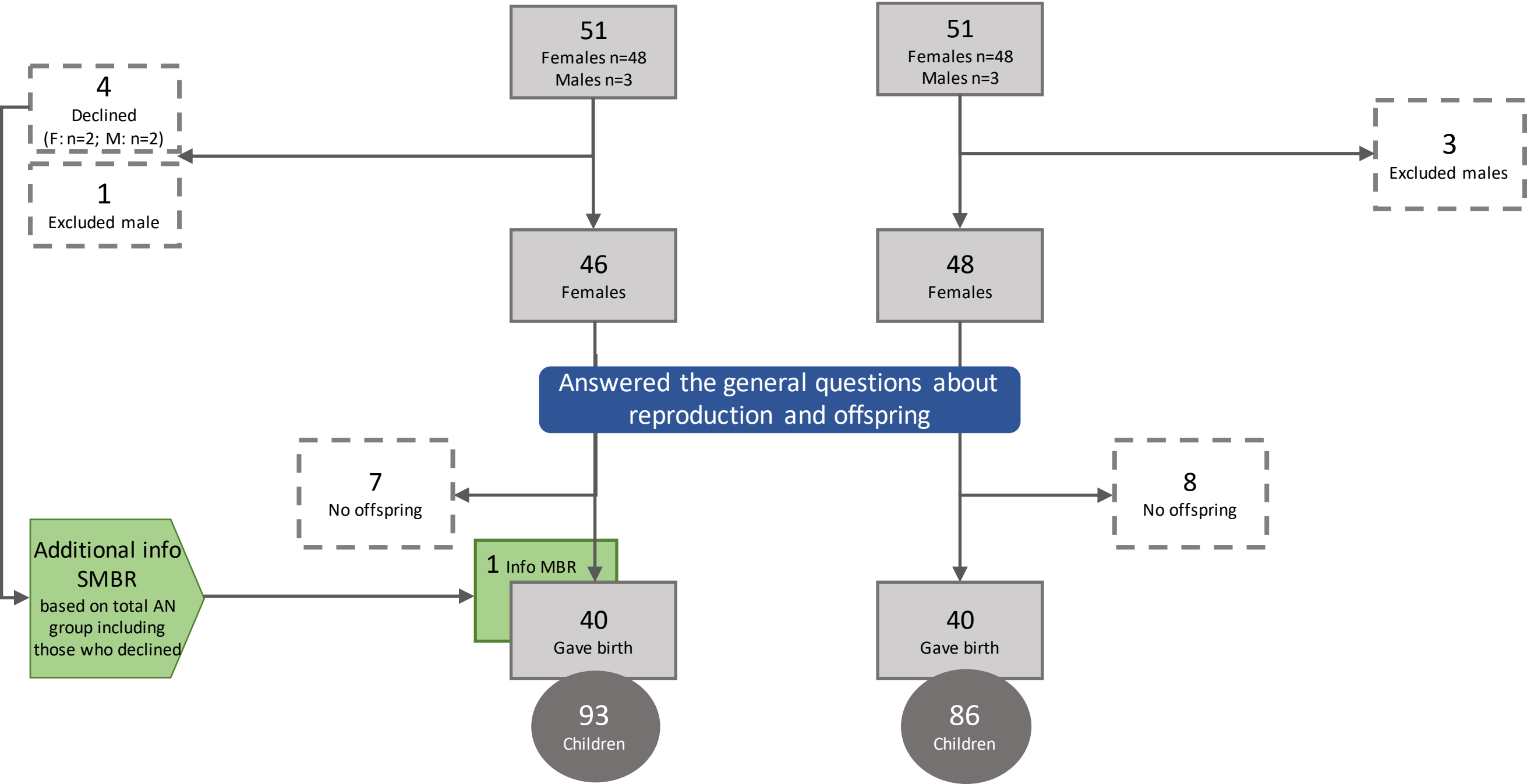

Supplement: Supplementary file 1 — Supplementary file1 Fig. S1. Participant flowchart showing original and final sample. Regarding the two females who declined; one had offspring and one had no offspring according to data from the SMBR. AN: Anorexia nervosa; COMP: Comparison; SMBR: the Swedish medical birth register; F: female; M: male. (PDF 27 KB) [file 787_2024_2393_MOESM1_ESM.pdf]
